# Female philopatry in a heterogeneous environment: ordinary conditions leading to extraordinary ESS sex ratios

**DOI:** 10.1186/1471-2148-7-13

**Published:** 2007-02-06

**Authors:** Vincent Hulin, Jean-Michel Guillon

**Affiliations:** 1 Univ Paris-Sud, CNRS, AgroParisTech, Laboratoire Ecologie, Systématique et Evolution, UMR 8079, Bâtiment 362, Orsay, F-91405, France

## Abstract

**Background:**

We use a simulation-based model to study the impact of female philopatry and heterogeneity of habitat quality on the evolution of primary sex ratio.

**Results:**

We show that these conditions may lead to strongly biased ESS habitat-dependent sex ratios, under two kinds of density-dependent population regulation. ESS sex ratios are always biased towards females in good habitats, towards males in poor habitats, and are generally equilibrated considering the whole population. Noticeably, the predicted bias of sex ratio usually increases with decreasing female philopatry.

**Conclusion:**

The selection forces responsible for these results are fully described. This study provides a new perspective on the evolutionary significance of temperature sex determination. We discuss the case of turtles by comparing our theoretical results with field observations.

## Background

Natal philopatry, i. e. the tendency for individuals to breed at or near their place of origin, has been described in a variety of animal species, including mammals [1], birds [reviewed in [2]], reptiles [3], and fish [4]. In such species, sex biased dispersal has often been observed as a result of natal homing being more frequent in one sex than in the other [5,6]. Indeed, there seems to be a tendency for female-biased dispersal in birds and male-biased dispersal in mammals [7-9].

Sex-biased dispersal has important consequences on the dynamics and on the social and genetic structures of natural populations [10,11], as well as on the evolution of phenotypic traits [12]. In particular, sex-biased dispersal provides the conditions for the evolution of biased sex ratios: parental manipulation of the sex ratio allows individuals to avoid kin competition [13,14], to benefit from local resource enhancement [15], or to select habitat in a heterogeneous environment [16].

Since its description, the determination of sex by temperature (TSD) in many reptiles has been a long standing puzzle from an evolutionary point of view [17-21]. The extreme sex ratios sometimes found in natural nests are indeed difficult to reconcile with the Fisherian frequency-dependent selection for equal investment in both sexes [22]. In order to find an adaptive explanation for environmental sex determination, Charnov and Bull [23] proposed a theoretical model in which habitat is heterogeneous and sexes benefit differentially from habitat quality. However, according to Warner and Shine [24], the assumptions of this model are difficult to test in reptiles, and the published literature "reflects its overall plausibility [...] rather than specific experimental evidence".

One of the latest proposed hypotheses accounting for TSD in reptiles is based on female natal philopatry [16,25]. In sea turtles in particular, there is substantial evidence for natal homing of nesting females [26,27] and for the existence of male dispersal [28-30]. This has lead to the hypothesis that nest site quality, if incubation success differs between nesting sites, could be inherited maternally [31]. Such spatial variability of incubation success is apparently frequent on nesting grounds [32-34].

According to Reinhold [25] and Julliard [16], sex-specific dispersal should lead to satisfy Charnov and Bull's [23] assumption that sexes benefit differentially from habitat quality. In a heterogeneous environment, natural selection should favour the sex ratio strategy maximizing the number of offspring breeding in high-quality habitats. Therefore, the evolutionary stable strategy (ESS) of sex ratio is one that overproduces the less dispersing sex (females in the case of female natal homing) in high-quality nesting sites and overproduces the most dispersing sex (males in the case of female natal homing) in low-quality nesting sites [16].

The model of Julliard [16] is based on several assumptions other than sex-biased dispersal and habitat patches of different quality. First, it assumes that reproduction, from mating to birth, occurs in the same patch. It also assumes that the population size is regulated by density-dependence occurring within each patch. These assumptions may be violated in migrating species, such as aquatic turtles that live in water and nest on earth. Because the scales at which mating and density-dependent regulation occur are key factors for population dynamics and evolution [35], we here present a new model introducing important modifications: mating sites are independent from nesting sites, and population regulation may occur either within nesting sites (hereafter named HABITAT model) or at the level of the whole population (TOTAL model). In any case, we show that the ESS primary sex ratio can be strongly biased depending on the nesting habitat but that the sex ratio of the overall population is generally equilibrated.

## Results

### The model

**Figure 1:**
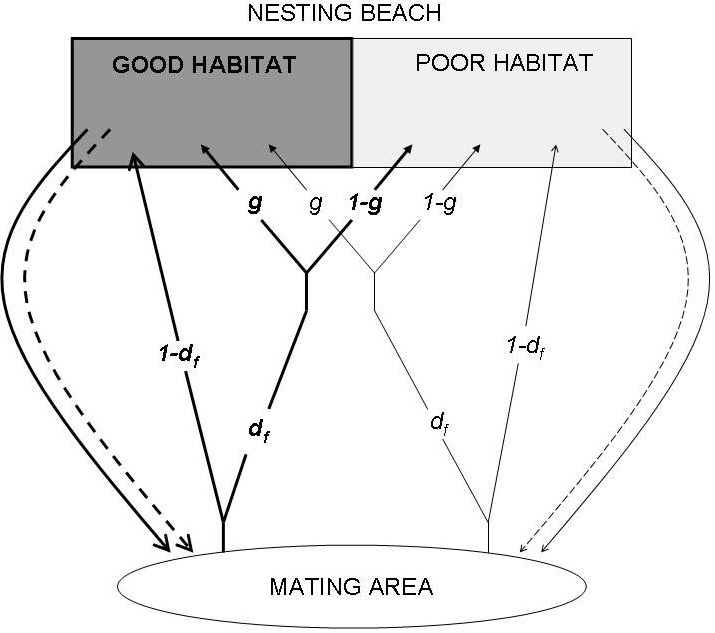
**Simplified life-cycle used in the model**. The parameters on the lines are the probability for an individual to follow it (equal to 1 in the absence of notation). Dashed lines are for males, plain lines for females. Bold lines represent individuals native from GOOD habitats, thin lines represent individuals native from POOR habitats.

We use an individual-based simulation model to find the ESS primary sex ratio strategy dependent on habitat quality under female natal philopatry. The model describes a simplified life-cycle of sea turtles (fig. 1).

#### Nesting beach

The nesting beach is divided in 2 kinds of habitat differing in their quality: GOOD habitats (proportion *g *of the nesting beach) or POOR habitats (proportion 1-*g*), with 0 <* g *< 1 (fig. 1). In GOOD habitats, a nesting female produces *F *times more offspring than in POOR habitats, with *F *> 1.

#### Sex ratio

We use a genetic architecture that allows the unconstrained evolution of sex ratio in each habitat so that the ESS is reached at the equilibrium. The strategy of sex ratio related to the habitat for every adult is determined by 2 alleles (*G*_1_/*G*_2_) the mean of which determines the offspring sex ratio (percentage of males) for nests in GOOD habitats, and 2 alleles (*P*_1_/*P*_2_) the mean of which determines the sex ratio for nests in POOR habitats, with *G*_1_, *G*_2_, *P*_1 _and *P*_2_, between 0 and 1. These alleles are located on 2 unlinked loci so that any offspring independently inherits one allele of its mother (*G*_*mother *_and *P*_*mother*_) and one of its father (*G*_*father *_and *P*_*father*_) at each locus. At each generation, an allele has a probability 0.005 to mutate, and one mutation is an increase or decrease of 0.005 in the value of the allele.

#### Population

The population size is fixed equal to 5,000 adults. Each adult is defined by its sex (male or female), the kind of habitat where it was born (GOOD or POOR), and the values of *G*_1_, *G*_2_, *P*_1 _and *P*_2_. Generations are discrete: adults breed once before dying. The sex ratio of the overall population (*SR*_*tot*_) is defined as the total number of males divided by 5,000.

#### Reproduction and dispersal

Mating takes place in a unique reproductive area (fig. 1) where all adults meet, regardless of their provenance habitat. Each female mates with one randomly chosen male. Females then return to the beach to nest. A proportion (1-*d*_*f*_) of the females ('non-dispersing females') nest in the same kind of habitat where they were born. The complement (*d*_*f*_) are considered as 'dispersing females' and are randomly distributed between GOOD and POOR habitats: for any dispersing female, the probability to nest in a GOOD habitat is *g *and in a POOR habitat is (1-*g*).

#### Density-dependence regulation

We apply one of two different kinds of density-dependent regulation. The first one (called HABITAT) occurs in each habitat and corresponds to a regulation at the scale of the nesting beach: 5,000 individuals will grow to adulthood, a proportion *Fg*/(*Fg+*1-*g*) born in GOOD habitats and a proportion (1-*g*)/(*Fg*+1-*g*) born in POOR habitats. The second one (called TOTAL) consists in the random draw of 5,000 individuals in the entire population of offspring, which will grow into adulthood. Then, in the adult population, the proportions of individuals born in GOOD habitats and in POOR habitats are respectively *FN*_*g*_/(*FN*_*g*_+*N*_*p*_) and *N*_*p*_/(*FN*_*g*_+ *N*_*p*_), with *N*_*p *_and *N*_*g *_the numbers of females nesting in POOR and GOOD habitats. This corresponds to a regulation at the scale of the entire population, on feeding grounds for example.

### Simulation results

#### HABITAT density-dependent regulation (fig. 2)

**Figure 2:**
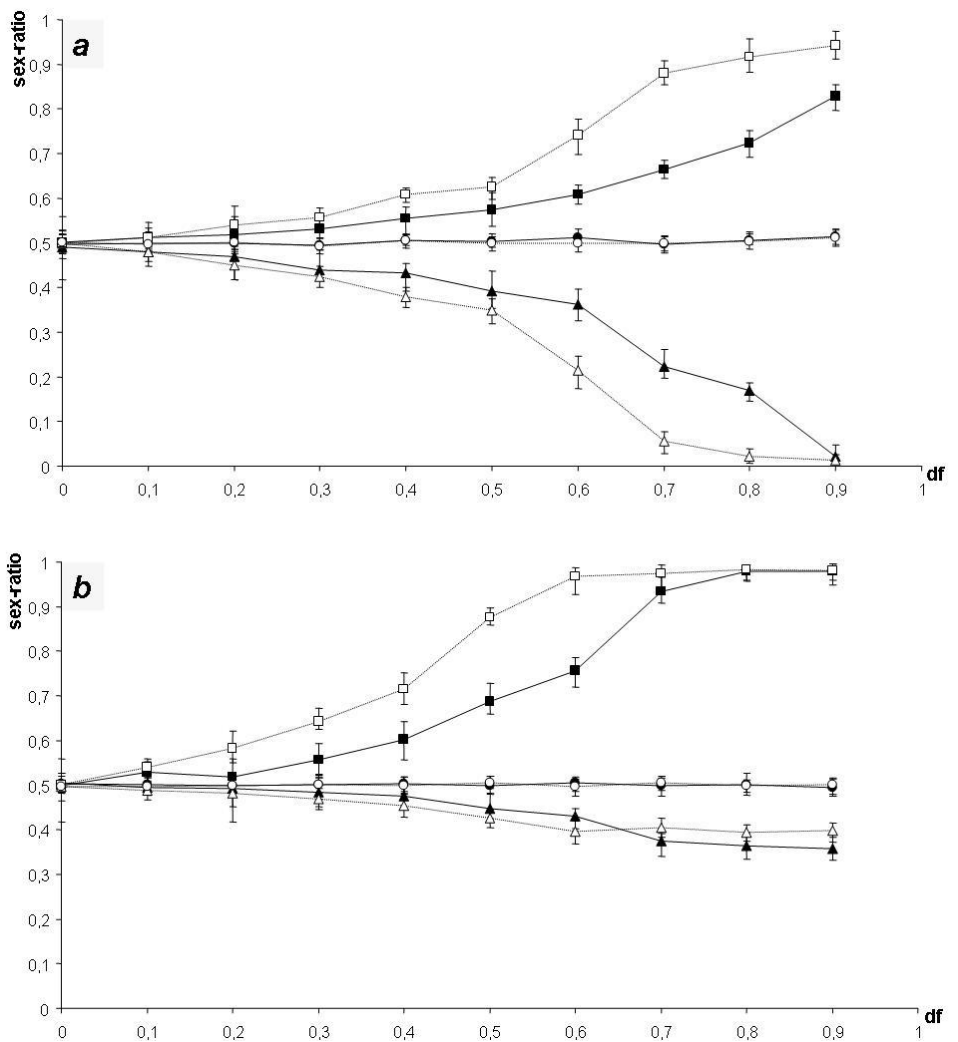
**ESS sex ratios in GOOD habitats (*G*), POOR habitats (*P*) and in the whole population (*SR*_*tot*_) as a function of female dispersal rate (*d*_*f*_) in the model with habitat density-dependent regulation. (a): *g *= 0.3. (b): *g *= 0.7**. Bars show maximal and minimal values in 20,000 generations at the equilibrium. Triangles: *G*, squares: *P *and circles: *SR*_*tot*_. Plain symbols: *F *= 1.5, open symbols: *F *= 2. Results are shown for simulations run with initial allele values of *G*_1_,*G*_2_,*P*_1 _and *P*_2 _= 0.5.

For *d*_*f *_= 0 (total philopatry), the ESS sex ratios are equilibrated in both habitats (*G *= *P *= 0.5). For 0 <* d*_*f *_< 1 (partial philopatry), the sex ratio is always biased towards males in the POOR habitat and towards females in the GOOD habitat (*G *< 0.5 <*P*). For given values of *F *and *d*_*f*_, the sex ratio is more biased in the habitat that contributes less to the whole population: when the proportion of females nesting in GOOD habitats is higher than the proportion of females nesting in POOR habitats (*Fg *> 1-*g*), the sex ratio is more biased in the POOR habitat; when *Fg *< 1-*g*, the sex ratio is more biased in the GOOD habitat. The sex ratio of the whole population is always unbiased (*SR*_*tot *_= 0.5). When *F *or *d*_*f  *_increases, the habitat-dependent sex ratios are more and more biased, until the sex ratio in one habitat may become totally biased (*P *= 1 or *G *= 0). This is illustrated in fig. 2b by *g *= 0.7, *F *= 2 and *d*_*f  *_= 0.9.

#### TOTAL density-dependent regulation (fig. 3)

**Figure 3:**
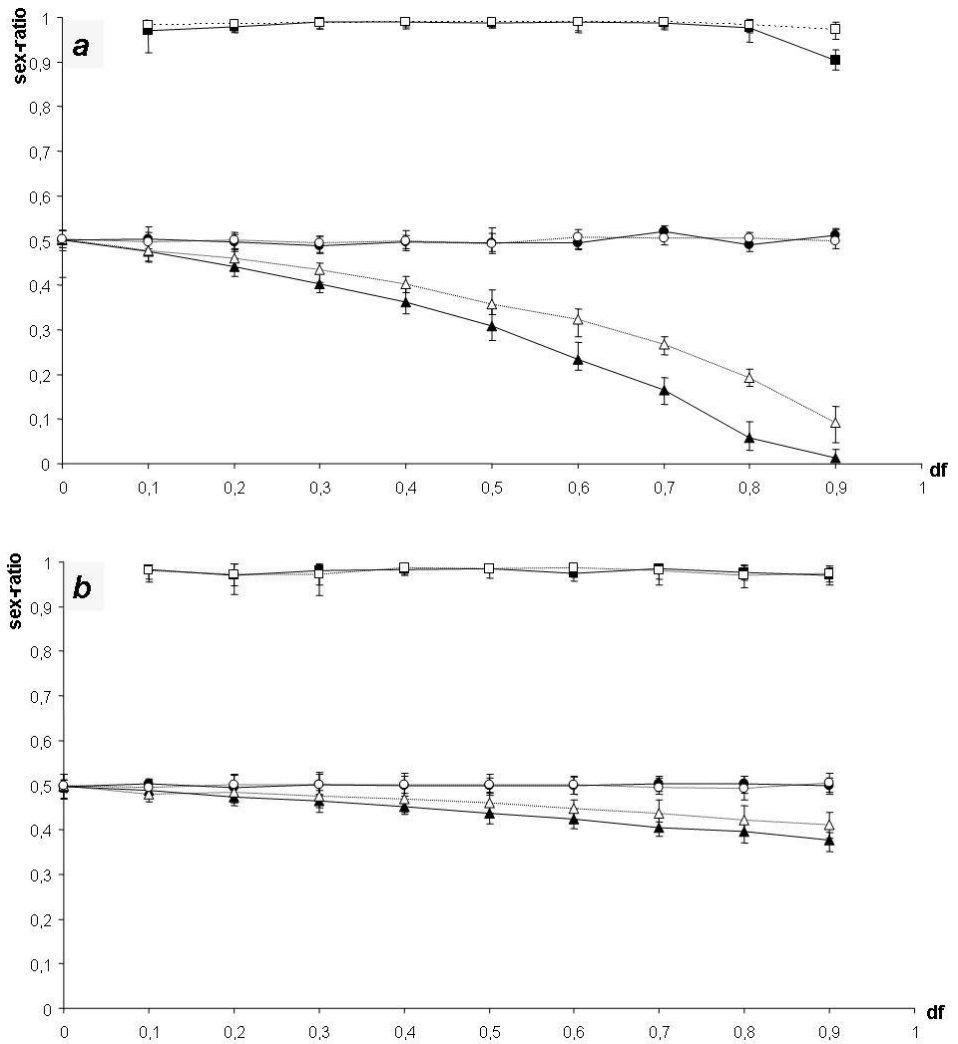
**ESS sex ratios in GOOD habitats (*G*), POOR habitats (*P*) and in the whole population (*SR*_*tot*_) as a function of female dispersal rate (*d*_*f*_) in the model with total density-dependent regulation. (a): *g *= 0.3. (b): *g *= 0.7**. Bars show maximal and minimal values in 20,000 generations at the equilibrium. Triangles: *G*, squares: *P*, and circles: *SR*_*tot*_. Plain symbols: *F *= 1.4, open symbols: *F *= 2. Results are shown for simulations run with initial allele values of *G*_1_,*G*_2_,*P*_1 _and *P*_2 _= 0.5.

For *d*_*f *_= 0 (total philopatry), females nest only in GOOD habitats and the situation is the same as with a single population nesting in a homogeneous environment (*G *= *SR*_*tot *_= 0.5). For 0 <* d*_*f  *_< 1 (partial philopatry), the sex ratio is always biased towards males in POOR habitats and towards females in GOOD habitats (*G *< 0.5 <*P*) like in the HABITAT model. For low values of *d*_*f*_, only males are produced in POOR habitats (*P *= 1) and the sex ratio in GOOD habitats is such that *SR*_*tot *_= 0.5. In contrast with the HABITAT model (i) as soon as *d*_*f *_> 0, the sex ratio is here totally male biased in POOR habitats, even for low values of *F*, and (ii) as long as *F *> 1, a decrease of *F *leads the sex ratio in GOOD habitats to be more female biased. When *d*_*f *_increases, the sex ratio in GOOD habitats is more and more female biased until it may become totally biased (*G *= 0) as well. When *G *= 0, a further increase of *d*_*f *_leads the sex ratio in POOR habitats to decrease (*P *< 1) and *SR*_*tot *_to increase (*SR*_*tot *_> 0.5). This is illustrated in fig. 3a by *g *= 0.3, *F *= 1.4 and *d*_*f *_= 0.9.

## Discussion

### Interpretation of the results and comparison with previous models

From our simulation results, we identify 2 evolutionary forces leading to the ESS sex ratios in our models. The first force is the consequence of mating taking place in a unique area for the entire population, leading *SR*_*tot *_to be equal to 0.5 [22]. The second force (habitat selection) is due to the difference in quality between habitats and the difference in dispersal rate between sexes: because females are always the less dispersing sex unless *d*_*f *_= 1, female offspring should be under-produced in POOR habitat and overproduced in GOOD habitats in order to increase the likelihood that females will nest in GOOD habitat [16].

In the HABITAT model, in case of high female philopatry (low *d*_*f *_values), the overproduction of female offspring in GOOD habitats may lead to a high number of females returning in GOOD habitats to nest, and thus to a higher competition for resources in GOOD habitats compared to POOR habitats. In the case of low female philopatry (high *d*_*f *_values), nesting females are more evenly distributed between habitats, and the strength of the competition for resources in GOOD habitats decreases. For a given value of *d*_*f*_, the optimal distribution of adults between habitats (i.e. when the competition for resources is equal between habitats) is attained when there are *F *times more females nesting in GOOD habitats than females nesting in POOR habitats (ideal free distribution of nests [36]). For *d*_*f *_= 0, this is obtained with unbiased sex ratios (*G *= *P *= 0.5). For *d*_*f *_> 0, the female bias in GOOD habitats increases with higher values of *d*_*f *_in order to reach the ideal free distribution of nests. When *F *increases, GOOD habitats can receive more females per unit of resource, and the sex ratio is then more biased towards females in GOOD habitats.

To sum up, a strategy of sex ratio must fulfil two conditions to be an ESS in the HABITAT model: (i) The sex ratio of the whole population is equilibrated; (ii) The number of nesting females per unit of resource is *F *times larger in GOOD habitats than in POOR habitats. These two conditions derived from our verbal argument can be expressed mathematically as:

(i)SRtot=0.5⇔Fg(1−G)+(1−g)(1−P)Fg+(1−g)=0.5
 MathType@MTEF@5@5@+=feaafiart1ev1aaatCvAUfKttLearuWrP9MDH5MBPbIqV92AaeXatLxBI9gBaebbnrfifHhDYfgasaacH8akY=wiFfYdH8Gipec8Eeeu0xXdbba9frFj0=OqFfea0dXdd9vqai=hGuQ8kuc9pgc9s8qqaq=dirpe0xb9q8qiLsFr0=vr0=vr0dc8meaabaqaciaacaGaaeqabaqabeGadaaakeaafaqabeqacaaabaWaaeWaaeaacqqGPbqAaiaawIcacaGLPaaaaeaacqWGtbWucqWGsbGudaWgaaWcbaGaemiDaqNaem4Ba8MaemiDaqhabeaakiabg2da9iabicdaWiabc6caUiabiwda1iabgsDiBpaalaaabaGaemOrayKaem4zaC2aaeWaaeaacqaIXaqmcqGHsislcqWGhbWraiaawIcacaGLPaaacqGHRaWkdaqadaqaaiabigdaXiabgkHiTiabdEgaNbGaayjkaiaawMcaamaabmaabaGaeGymaeJaeyOeI0IaemiuaafacaGLOaGaayzkaaaabaGaemOrayKaem4zaCMaey4kaSYaaeWaaeaacqaIXaqmcqGHsislcqWGNbWzaiaawIcacaGLPaaaaaGaeyypa0JaeGimaaJaeiOla4IaeGynaudaaaaa@59A4@

(ii)NgFg=Np1−g⇔1−P1−G=F(Fgdf−1+df−gdf)(1−g)df−F(1−gdf)
 MathType@MTEF@5@5@+=feaafiart1ev1aaatCvAUfKttLearuWrP9MDH5MBPbIqV92AaeXatLxBI9gBaebbnrfifHhDYfgasaacH8akY=wiFfYdH8Gipec8Eeeu0xXdbba9frFj0=OqFfea0dXdd9vqai=hGuQ8kuc9pgc9s8qqaq=dirpe0xb9q8qiLsFr0=vr0=vr0dc8meaabaqaciaacaGaaeqabaqabeGadaaakeaafaqabeqacaaabaWaaeWaaeaacqqGPbqAcqqGPbqAaiaawIcacaGLPaaaaeaadaWcaaqaaiabd6eaonaaBaaaleaacqWGNbWzaeqaaaGcbaGaemOrayKaem4zaCgaaiabg2da9maalaaabaGaemOta40aaSbaaSqaaiabdchaWbqabaaakeaacqaIXaqmcqGHsislcqWGNbWzaaGaeyi1HS9aaSaaaeaacqaIXaqmcqGHsislcqWGqbauaeaacqaIXaqmcqGHsislcqWGhbWraaGaeyypa0ZaaSaaaeaacqWGgbGrdaqadaqaaiabdAeagjabdEgaNjabdsgaKnaaBaaaleaacqWGMbGzaeqaaOGaeyOeI0IaeGymaeJaey4kaSIaemizaq2aaSbaaSqaaiabdAgaMbqabaGccqGHsislcqWGNbWzcqWGKbazdaWgaaWcbaGaemOzaygabeaaaOGaayjkaiaawMcaaaqaamaabmaabaGaeGymaeJaeyOeI0Iaem4zaCgacaGLOaGaayzkaaGaemizaq2aaSbaaSqaaiabdAgaMbqabaGccqGHsislcqWGgbGrdaqadaqaaiabigdaXiabgkHiTiabdEgaNjabdsgaKnaaBaaaleaacqWGMbGzaeqaaaGccaGLOaGaayzkaaaaaaaaaaa@6A81@

with *N*_*p *_and *N*_*g *_the number of female nesting in POOR and GOOD habitats, respectively. When the second condition leads the sex ratio in POOR habitats to be totally male biased (*P *= 1), the sex ratio in GOOD habitats is determined by the first condition (*SR*_*tot *_= 0.5). This case is illustrated in fig. 2b for *g *= 0.7, *F *= 2 and *d*_*f *_= 0.8. When the second condition leads the sex ratio in GOOD habitats to be totally female biased (*G *= 0), the Fisherian force still favours an unbiased sex ratio for the entire population (*SR*_*tot *_= 0.5) while the habitat selection force favours the production of more males in POOR habitats. The two selective forces then equilibrate for *G *= 0 and for an intermediate value of *P*, with *P *> 0.5 and *SR*_*tot *_> 0.5. The sex ratio of the whole population is male-biased but stays close to 0.5 (results not shown).

In the TOTAL model, the first force, conducting *SR*_*tot *_to be unbiased, is the same as in the HABITAT model. However, the habitat selection force is different: because there is no density-dependent regulation in habitats, a nest in a POOR habitat always produces *F *times fewer adults in the next generation than a nest in a GOOD habitat. So, it is always more advantageous for females to nest in GOOD habitats. Whatever the values of *d*_*f *_and *g*, the probability to nest in GOOD habitats is higher for females born in GOOD habitats than for females born in POOR habitats. Consequently, females should be produced in GOOD habitats rather than in POOR habitats. For males, regardless of the habitat where they are born, the probability of mating with a female that will nest in a GOOD habitat is the same. Hence, the second force selects against the production of females in POOR habitats, resulting in the production of males only. In GOOD habitats, the ESS sex ratio is the one that permits *SR*_*tot *_to be equilibrated. These two conditions derived from our verbal argument can be expressed mathematically as:

(i)     *P *= 1

(ii)SRtot=0.5⇔FNg(1−G)FNg+Np=0.5⇔G=0.5(1−df(1−g)F(1−df+gdf))
 MathType@MTEF@5@5@+=feaafiart1ev1aaatCvAUfKttLearuWrP9MDH5MBPbIqV92AaeXatLxBI9gBaebbnrfifHhDYfgasaacH8akY=wiFfYdH8Gipec8Eeeu0xXdbba9frFj0=OqFfea0dXdd9vqai=hGuQ8kuc9pgc9s8qqaq=dirpe0xb9q8qiLsFr0=vr0=vr0dc8meaabaqaciaacaGaaeqabaqabeGadaaakeaafaqabeqacaaabaWaaeWaaeaacqqGPbqAcqqGPbqAaiaawIcacaGLPaaaaeaacqWGtbWucqWGsbGudaWgaaWcbaGaemiDaqNaem4Ba8MaemiDaqhabeaakiabg2da9iabicdaWiabc6caUiabiwda1iabgsDiBpaalaaabaGaemOrayKaemOta40aaSbaaSqaaiabdEgaNbqabaGcdaqadaqaaiabigdaXiabgkHiTiabdEeahbGaayjkaiaawMcaaaqaaiabdAeagjabd6eaonaaBaaaleaacqWGNbWzaeqaaOGaey4kaSIaemOta40aaSbaaSqaaiabdchaWbqabaaaaOGaeyypa0JaeGimaaJaeiOla4IaeGynauJaeyi1HSTaem4raCKaeyypa0JaeGimaaJaeiOla4IaeGynauZaaeWaaeaacqaIXaqmcqGHsisldaWcaaqaaiabdsgaKnaaBaaaleaacqWGMbGzaeqaaOWaaeWaaeaacqaIXaqmcqGHsislcqWGNbWzaiaawIcacaGLPaaaaeaacqWGgbGrdaqadaqaaiabigdaXiabgkHiTiabdsgaKnaaBaaaleaacqWGMbGzaeqaaOGaey4kaSIaem4zaCMaemizaq2aaSbaaSqaaiabdAgaMbqabaaakiaawIcacaGLPaaaaaaacaGLOaGaayzkaaaaaaaa@704A@

with *N*_*p *_and *N*_*g *_the numbers of females nesting in POOR and GOOD habitats, respectively. When *F *increases, GOOD habitats produce more individuals compared to POOR habitats, so the ESS sex ratio in GOOD habitats needs to be less female biased to equilibrate the global sex ratio.

When it is not possible to satisfy these two conditions simultaneously (i.e. when *F *<* d*_*f*_(1 - *g*)/(1-*d*_*f*_+*gd*_*f*_)), the habitat selection force still favours a totally male-biased sex ratio in POOR habitats, while the Fisherian force favours the production of some females in POOR habitats so that *SR*_*tot *_= 0.5. The two selective forces then equilibrate for *G *= 0 and for an intermediate value of *P*, with *P *< 1 and *SR*_*tot *_> 0.5. The sex ratio of the whole population is male-biased but stays close to 0.5. This case is illustrated in fig. 3b for *g *= 0.3, *F *= 2 and *d*_*f *_= 0.9.

With either kind of density-dependent regulation, our results show that partial female philopatry (0 <* d*_*f *_< 1) leads the ESS sex ratio to be biased towards males in POOR habitats and towards females in GOOD habitats. Extremely biased sex ratios are obtained for higher values of *F *and *d*_*f *_in our HABITAT model and most values of *d*_*f *_and *F *in our TOTAL model. We predict extraordinary sex ratios for ordinary values of parameters, especially in the TOTAL model where only males may be produced in POOR habitats. These conditions are likely to be met in many situations involving female philopatry, including the case of sea turtles (see below). The population size assumed in our model is quite large and the population is panmictic. Therefore, the selective forces resulting from kin competition (Local Mate Competition and Local Resource Competition [13,37]) have no influence.

The density-dependent regulation in our HABITAT model is the same as in Julliard [16]. However, here both sexes migrate before mating in an area distinct from nesting habitats. The results of Julliard's model and ours are similar on 2 points: (i) ESS sex ratios are male-biased in POOR habitats and female-biased in GOOD habitats, and (ii) the bias of ESS sex ratio increases when the female philopatry decreases. In contrast with Julliard, we find an unbiased ESS sex ratio for the overall population. Guillon *et al. *[38] have refined the model of Julliard [16] by calculating reproductive values in a more comprehensive way. Total male dispersal (*d*_*m *_= 1) in their model yields the same results as our HABITAT model, although the life cycles modelled are indeed different.

A promising model by Reinhold [25] has already proposed that female philopatry and spatial heterogeneity offer the conditions for the evolution of environmental sex determination in reptiles. This study assumed the same global density-dependent regulation as in our TOTAL model and concluded that a sex ratio strategy biased towards males in low-quality sites and towards females in high-quality sites was favoured relatively to unbiased sex ratios resulting from genetic sex determination. The method used by Reinhold did not allow him to find the values for the ESS sex ratios, yet his results suggested that the sex ratio was equilibrated at the whole population scale. Reinhold [25] also restricted the range of his parameters : (i) high-quality habitats were assumed to be rare (equivalent in our model to *g *< 0.5), and (ii) the proportion of females born in low-quality sites but nesting in high-quality sites was constrained by the difference in habitat quality (equivalent to *Fd*_*f*_(1 - *g*) <* F *- 1 in our model, i.e. high female philopatry or high difference in habitat quality). We here show that biased sex ratios strategies can invade and get to fixation beyond Reinhold's range of parameters. Indeed, low *F *and high *d*_*f *_values are biologically realistic and give the most extreme sex ratios in our study, these results being quite unexpected. Furthermore, we obtain the values for the ESS sex ratio and show why equilibrated population sex ratio is a necessary condition for ESS in most cases. In contrast, Freedberg and Wade [31] have proposed that inheritance of nest-site through female philopatry could lead to female biased sex-ratio at the level of the whole population. Their conclusion was not based on an ESS analysis and is therefore difficult to compare to our results.

### Implications for the evolution of TSD in reptiles

The model may apply to any species with Environmental Sex Determination or with maternal control of sex allocation that fits our main assumptions, namely heterogeneity of habitat quality and female philopatry. The case of sea turtles, which is probably the most documented one, is discussed below.

The first key assumption of the model is that the environment is heterogeneous with respect to survival from oviposition to reproduction. The model then predicts that the primary sex ratio should adjust to the quality of the nesting environment, with more females being produced at high quality habitats and more males at low quality habitats. For species where females are produced at high incubation temperature (TSD Ia), this would be the case if temperature during incubation positively correlates with nest success. Heterogeneity in temperature has often been described between neighbouring nesting beaches, due to difference in composition or albedo of the sand [e.g., [32,39]]. Temperature heterogeneity can also be found within a nesting beach. The cooling effect of tides creates a decrease of temperature from higher to lower beach zone [34,40-42], and the back of the beach may be cooler than the open beach, due to the presence of shadowing vegetation [43,44]. Interestingly, low temperature beaches or zones are often associated with a relatively lower hatching success [[32,34,39,41,42,45], but see [46]]. Indeed, nests on the lower beach can be lost due to erosion or inundation [33,34,47-52], and nests in the vegetation zone may suffer a higher predation rate or rupture risk [53-55]. In addition, nests in the lower beach zone may be more at risk of inundation by rainfall [56,57] and hatchlings emerging in the vegetation zone may face orientation problems in finding the sea [49,58,59]. Low temperature itself could influence hatching success by slowing the development of embryos and thus increase incubation time and thereby the risk of loss, destruction or predation. Overall, on many nesting grounds, even though excessively high temperatures can have detrimental effects on incubation process [60], a higher nest success could correlate with relatively high, feminizing, temperatures, as predicted by the model.

The present model investigates the consequences of female philopatry on the ESS sex ratio. Adult natal philopatry is difficult to observe in species with delayed sexual maturity, such as sea turtles because of the long time between birth and the first reproduction event. Nevertheless, the use of maternally inherited genetic markers (mitochondrial DNA) has provided support for female natal homing at a regional scale [e.g., [61-64]]. At a finer spatial scale, genetic isolation by distance of female green turtles has been observed on the beach of Tortuguero [65]. In addition, nest site fixity, i.e. the tendency for an individual female to cluster its nests, has been observed within a given season (renesting events) at the scale of different beaches [e.g., [48,49,66-68]], along the coastal axis of a nesting beach [e.g., [26,44,69]] or along the vegetation to ocean axis [44,59]. The same behaviour has also been observed for female sea turtles nesting in several breeding seasons (remigration events) [26,28,48,67,70]. Overall, although female sea turtles seem to be highly philopatric to their natal region, further work is still needed to test the model's predictions. In this aim, studies of female philopatry in relation with spatial variation of nesting success and sex ratios would be greatly valuable.

Destruction of previous nests by nesting females has been observed on several beaches [71,72]. Caut *et al. *[73] have shown that such a covering of nests may also be detrimental to the incubation success of the overlaying nest. At saturation, the incubation success of the nesting area is expected to tend to a finite rate, depending on the carrying capacity of the laying environment. This is the basis for the density-dependent regulation assumed in our HABITAT model. Such a saturation may be rarely observed now, given the important human pressure on sea turtle populations in the recent years by egg poaching, turtle hunting and accidental catching [74], but could have been reached in the past when sea turtles were much more abundant. Alternatively, populations could be regulated at the sea, by predation on juveniles or by competition for food, as described in our TOTAL model. If density-dependent regulation occurs at both levels (first on nesting beaches and then at sea), the evolution of sex ratio should follow the same pattern as in the case of HABITAT density-dependent regulation alone. Indeed, this would modify the HABITAT model only by adding a random draw of individuals from the adult population.

The model's assumptions may be satisfied in other species of turtles. In freshwater turtles, nest temperature could be positively correlated with hatching success [[75-77], but see [78]]. Female freshwater turtles exhibit nest site-fidelity [e.g., [79-82]]. Furthermore, molecular studies have found significant genetic structure among nearby nesting sites [83,84] or genetic isolation by distance within a nesting site [82], suggesting that natal homing is present in freshwater turtles too.

### Perspectives for refining the model

An important feature of the model is panmixia, resulting from the absence of male philopatry. This assumption may be violated in a variety of species. Further modelling is warranted to investigate the consequences of relaxing the hypothesis of panmixia, but preliminary work indicates that the predicted sex ratios are very similar as long as females are more philopatric than males.

In the present model, generations are discrete; i. e. individuals reproduce only once before dying. Describing a long-lived species, with a juvenile phase and multiple reproductive episodes, is not expected to change the predictions of the model. Only the time needed to reach the ESS should increase [85]. However, introducing a temporally variable environment is expected to change the predictions of the model, especially in the case of overlapping generations. The intensity of the habitat selection force should decrease as the habitat becomes less predictable from one generation to the next. Further work would be useful to study the influence of temporal variation of habitat quality on the ESS sex ratios.

Another improvement of the model could be to allow females to prefer high quality sites. In the HABITAT model, perfect habitat selection by dispersing females (a GOOD habitat is chosen *F *times more often than a POOR habitat), leads to an ideal free distribution of breeding females. This should cancel the advantage of sex ratio biasing [16,38]. In contrast, unless *d*_*f *_= 1, perfect habitat selection in the TOTAL model would not equalize the probabilities of different females reaching a GOOD habitat, and is thus not expected to yield equilibrate ESS sex ratios.

In our model, female dispersal can be considered as an imperfect philopatry resulting from constraints on orientation, migration or perception of the environment. Alternatively, dispersal could result from selection in a temporally variable environment: when the quality of the habitat is not completely predictable, individuals should adopt a strategy that permits them to explore other breeding-sites. It would thus be interesting to allow the joint evolution of sex allocation and dispersal rate [86].

## Conclusion

Our individual-based simulation model shows that female nest-site philopatry and heterogeneity of habitat quality provide sufficient conditions for the evolution of biased habitat-dependent sex ratios. In all cases, the evolutionary stable strategy is to overproduce females in good quality habitats and males in low quality habitats, while the sex ratio of the overall population is generally unbiased. The values for the ESS sex ratios are strongly dependent on the type of density-dependent regulation assumed. Highly biased sex ratios are predicted for biologically realistic values of parameters corresponding to low female philopatry and moderate difference in habitat quality.

To assess the contribution of our model in the study of the evolutionary significance of temperature-dependent sex determination, it should be tested in sea turtles by measuring sex ratios and incubation success of natural nests. We predict a positive correlation between incubation success, measured as the proportion of eggs yielding juveniles that reach the sea, and the proportion of females among hatchlings. In sea turtles, high temperatures during incubation lead to the overproduction of females in hatchlings. Preliminary evidence suggests that higher incubation success could be correlated with high (feminizing) temperatures. However, field studies are needed to obtain more convincing evidence.

## Methods

We search for the ESS values of sex ratio for different values of *F*, *g *and *d*_*f*_. At each generation *i*, we compute *G*^*i *^as the mean of alleles at the *G *locus in the adult population and *P*^*i *^as the mean of alleles at the *P *locus in the adult population. The total population sex ratio, SRtoti
 MathType@MTEF@5@5@+=feaafiart1ev1aaatCvAUfKttLearuWrP9MDH5MBPbIqV92AaeXatLxBI9gBaebbnrfifHhDYfgasaacH8akY=wiFfYdH8Gipec8Eeeu0xXdbba9frFj0=OqFfea0dXdd9vqai=hGuQ8kuc9pgc9s8qqaq=dirpe0xb9q8qiLsFr0=vr0=vr0dc8meaabaqaciaacaGaaeqabaqabeGadaaakeaacqWGtbWucqWGsbGudaqhaaWcbaGaemiDaqNaem4Ba8MaemiDaqhabaGaemyAaKgaaaaa@34D9@, is calculated as the number of males divided by 5,000 (the total number of adults). The simulations are run until values of *G*^*i*^, *P*^*i *^and SRtoti
 MathType@MTEF@5@5@+=feaafiart1ev1aaatCvAUfKttLearuWrP9MDH5MBPbIqV92AaeXatLxBI9gBaebbnrfifHhDYfgasaacH8akY=wiFfYdH8Gipec8Eeeu0xXdbba9frFj0=OqFfea0dXdd9vqai=hGuQ8kuc9pgc9s8qqaq=dirpe0xb9q8qiLsFr0=vr0=vr0dc8meaabaqaciaacaGaaeqabaqabeGadaaakeaacqWGtbWucqWGsbGudaqhaaWcbaGaemiDaqNaem4Ba8MaemiDaqhabaGaemyAaKgaaaaa@34D9@ are stable. We then compute *G*, *P *and *SR*_*tot*_, means of *G*^*i*^, *P*^*i *^and SRtoti
 MathType@MTEF@5@5@+=feaafiart1ev1aaatCvAUfKttLearuWrP9MDH5MBPbIqV92AaeXatLxBI9gBaebbnrfifHhDYfgasaacH8akY=wiFfYdH8Gipec8Eeeu0xXdbba9frFj0=OqFfea0dXdd9vqai=hGuQ8kuc9pgc9s8qqaq=dirpe0xb9q8qiLsFr0=vr0=vr0dc8meaabaqaciaacaGaaeqabaqabeGadaaakeaacqWGtbWucqWGsbGudaqhaaWcbaGaemiDaqNaem4Ba8MaemiDaqhabaGaemyAaKgaaaaa@34D9@, respectively, during 20,000 generations at the equilibrium. We take into account variations between generations by recording the maximum and minimum of *G*^*i*^, *P*^*i *^and SRtoti
 MathType@MTEF@5@5@+=feaafiart1ev1aaatCvAUfKttLearuWrP9MDH5MBPbIqV92AaeXatLxBI9gBaebbnrfifHhDYfgasaacH8akY=wiFfYdH8Gipec8Eeeu0xXdbba9frFj0=OqFfea0dXdd9vqai=hGuQ8kuc9pgc9s8qqaq=dirpe0xb9q8qiLsFr0=vr0=vr0dc8meaabaqaciaacaGaaeqabaqabeGadaaakeaacqWGtbWucqWGsbGudaqhaaWcbaGaemiDaqNaem4Ba8MaemiDaqhabaGaemyAaKgaaaaa@34D9@ during this period. For defined values of *F*, *g *and *d*_*f *_< 1, similar values are found for *G*, *P *and *SR*_*tot*_, regardless of the initial values of *G*_1_, *G*_2_, *P*_1 _and *P*_2_. In the absence of philopatry (*d*_*f *_= 1), depending on the simulation, we obtain different equilibrium values for sex allocations in GOOD and POOR habitats (*G*, *P*) such that *SR*_*tot *_= 0.5. Hence, results for *d*_*f *_= 1 are not presented in the figures.

## Authors' contributions

VH wrote the program, carried out the simulations and drafted the manuscript.

JMG participated in the design of the study and drafted the manuscript.

All authors read and approved the final manuscript.
